# Preoperative Immune-Related Adverse Events in Resectable NSCLC Treated With Neoadjuvant Nivolumab Plus Chemotherapy: A Multicenter Retrospective Analysis

**DOI:** 10.1016/j.jtocrr.2025.100930

**Published:** 2025-10-30

**Authors:** Takuya Watanabe, Kotaro Nomura, Shinkichi Takamori, Shinya Tane, Shuta Ohara, Hana Oiki, Shinya Katsumata, Makoto Endo, Satoshi Takamori, Marina Nakatsuka, Hironori Tenpaku, Ryuji Nakamura, Hirotsugu Notsuda, Kei Namba, Kentaro Minegishi, Masayuki Tanahashi, Masahiro Tsuboi, Junichi Soh, Mototsugu Shimokawa, Yasuhisa Ohde

**Affiliations:** a Division of Thoracic Surgery, Respiratory Disease Center, Seirei Mikatahara General Hospital, Hamamatsu, Japan; b Department of Thoracic Surgery, National Cancer Center Hospital East, Kashiwa, Japan; c Department of Surgery and Science, Graduate School of Medical Sciences, Kyushu University, Fukuoka, Japan; d Department of Thoracic and Breast Surgery, Oita University Faculty of Medicine, Oita, Japan; e Division of Thoracic Surgery, Kobe University Graduate School of Medicine, Kobe, Japan; f Division of Thoracic Surgery, Department of Surgery, Kindai University Faculty of Medicine, Osaka-Sayama, Japan; g Division of Thoracic Surgery, Shizuoka Cancer Center, Shizuoka, Japan; h Department of Thoracic Surgery, Yamagata Prefectural Central Hospital, Yamagata, Japan; i Department of Thoracic and Cardiovascular Surgery, Kuwana City Medical Center, Kuwana, Japan; j Department of Thoracic and Pediatric Surgery, Nagoya City University Graduate School of Medical Sciences, Nagoya, Japan; k Department of Thoracic Surgery, Institute of Development, Aging, and Cancer, Tohoku University, Sendai, Japan; l Department of Surgery, Okayama Rosai Hospital, Okayama, Japan; m Department of Thoracic Surgery, Saitama Medical Center, Jichi Medical University, Saitama, Japan; n Department of Thoracic Surgery, Osaka Metropolitan University Graduate School of Medicine, Osaka, Japan; o Department of Biostatistics, Graduate School of Medicine, Yamaguchi University, Ube, Japan

**Keywords:** Non–small cell lung cancer, Neoadjuvant therapy, Chemoimmunotherapy, Immune-related adverse events, Nivolumab

## Abstract

**Introduction:**

Preoperative immune-related adverse events (irAEs) during neoadjuvant chemoimmunotherapy for resectable non-small cell lung cancer (NSCLC) remain poorly characterized. We aimed to evaluate their frequency, clinical impact, and associated risk factors.

**Methods:**

This prespecified subanalysis of the multicenter CReGYT-04 Neo-Venus study retrospectively examined 130 patients with resectable NSCLC (stage IIA-IIIB, Union for International Cancer Control, eighth edition) treated with neoadjuvant nivolumab plus platinum-doublet chemotherapy. Clinical data were collected from 29 Japanese institutions. Patients were stratified according to the presence or absence of preoperative irAEs. Exploratory logistic regression was used to identify predictive factors.

**Results:**

Preoperative irAEs were observed in 18.5% of the patients (n = 24). Patients with irAEs had a significantly lower neoadjuvant therapy completion rate (58.3% versus 92.5%, *p* <0.001) and a higher incidence of cancelled surgery (21.7% versus 5.8%, *p* = 0.029) than those without irAEs. There were 18 patients (78.3%) with irAEs who underwent surgical resection. R0 resection was achieved in 94.4%. The postoperative complication rates and length of hospital stay were comparable between the groups. The major pathologic response rate (38.9% versus 63.1%) and pathologic complete response rate (27.8% versus 36.8%) were lower in patients with preoperative irAEs. Tumor size greater than 3.8 cm and eosinophil fraction greater than or equal to 2.0% were identified as exploratory predictors of preoperative irAEs.

**Conclusion:**

Preoperative irAEs occurred in approximately 20% of patients with resectable NSCLC treated with neoadjuvant nivolumab plus chemotherapy and were associated with treatment discontinuation and cancellation of surgery. Nevertheless, curative-intent surgery remained feasible and achieved acceptable perioperative outcomes in most patients with preoperative irAEs.

## Introduction

Neoadjuvant chemoimmunotherapy has emerged as a promising treatment strategy for resectable non-small cell lung cancer (NSCLC). The CheckMate 816 phase III trial reported that neoadjuvant nivolumab plus platinum-based chemotherapy substantial improved pathologic complete response and event-free survival relative to chemotherapy alone,^1^ leading to its adoption as a new standard of care. In Japan, neoadjuvant chemoimmunotherapy has been widely implemented since its approval, with a growing interest in understanding its impact in clinical practice.

Although perioperative chemoimmunotherapy has exhibited clinical benefits, immune-related adverse events (irAEs) remain a significant concern. In this context, the frequency of irAEs has been estimated to be approximately 10% to 20% on the basis of recent phase III trials.^1^, ^2^, ^3^ These trials did not specify whether irAEs occurred preoperatively or postoperatively, which made it difficult to estimate the frequency of preoperative irAEs. Furthermore, the extent to which irAEs affect the completion of neoadjuvant therapy or the feasibility of subsequent surgery has not been clearly reported. Most available data are derived from selected clinical trial populations, which may not fully reflect the patients typically seen in clinical practice. Consequently, the characteristics of patients who experience preoperative irAEs in clinical practice remain largely unknown.

To address these issues, we conducted a secondary analysis of the CReGYT-04 Neo-Venus study, the first multicenter registry in Japan, evaluating neoadjuvant chemoimmunotherapy for NSCLC. This subanalysis aimed to comprehensively characterize the clinical features, perioperative outcomes, and pathologic responses of patients who developed preoperative irAEs. We also explored the potential risk factors associated with their onset.

## Materials and Methods

### Study Design

This study was a prespecified subanalysis of the CReGYT-04 Neo-Venus study, a multicenter retrospective observational cohort study conducted at 29 institutions across Japan. The study period spanned from March 27, 2023 to July 31, 2024. The study protocol was approved by the institutional review board of the National Cancer Center (approval number: 2024-091). The requirement for written informed consent was waived owing to the retrospective nature of the study.

### Patient Selection

Patients were eligible for inclusion in the original cohort if they were 18 years of age or older, had resectable NSCLC at clinical stage IIA to IIIB on the basis of the eighth edition of the Union for International Cancer Control TNM classification, and received neoadjuvant therapy with nivolumab plus platinum-doublet chemotherapy during the study period. All patients were confirmed to have resectable disease at baseline, as determined by multidisciplinary evaluation before the initiation of neoadjuvant chemoimmunotherapy. After excluding one patient at the start of the first cycle, 130 patients were included in this subanalysis. All patients received at least one dose of neoadjuvant nivolumab. The patients were stratified according to the presence or absence of grade 1or higher preoperative irAEs.

### Data Collection

Clinical data were retrospectively collected from the electronic medical records at each participating institution. All data were anonymized and entered into a centralized electronic data capture system managed jointly by Yamaguchi University and National Cancer Center Hospital East.

Baseline all laboratory values were recorded at the initiation of the neoadjuvant chemoimmunotherapy. Clinical staging was determined according to the Union for International Cancer Control for TNM classification (eighth edition) on the basis of contrast-enhanced computed tomography, positron emission tomography–computed tomography, and pathologic evaluation of lymph nodes. N2 disease was further categorized as single-station or multistation involvement. Details of treatment-related adverse events, including the type, grade, and timing of irAEs, were extracted and graded according to the Common Terminology Criteria for Adverse Events (version 5.0). Data on the early discontinuation of neoadjuvant therapy, reasons for discontinuation, therapeutic interventions for irAEs, and surgical cancellation were also collected.

### Statistical Analyses

Baseline characteristics were compared between patients with and without preoperative irAEs. Categorical variables were analyzed using Fisher’s exact test, and continuous variables were assessed using the Mann-Whitney U test. Two-sided *p* values of less than 0.05 were considered to indicate statistical significance.

Univariate logistic regression analyses were conducted to explore clinical factors potentially associated with the development of preoperative irAEs. A multivariable analysis was not performed, as the number of events was expected to be limited. Continuous variables were dichotomized using median values. Odds ratios (ORs) and 95% confidence intervals (CIs) were also calculated.

All statistical analyses were performed using RStudio (ver. 4.3.2; RStudio Team, Boston, MA) and EZR (Saitama Medical Center, Jichi Medical University, Saitama, Japan).^4^

## Results

### Patient Characteristics

Of the 130 patients, 24 (18.5%) developed preoperative irAEs. The detailed types and severities of these irAEs are summarized in Table 1. The most frequently observed irAEs were rash, thyroid dysfunction (including hypothyroidism and thyrotoxicosis), and pneumonitis. Grade 3 or higher irAEs included pneumonitis, cytokine release syndrome, diabetes mellitus, and rashes.

**Table 1:** Types and Grades of Preoperative Immune-Related Adverse Events

Adverse event	Any grade	Grade ≥3
Rash	6	2
Pneumonitis	5	2
Hypothyroidism	5	2
Colitis	3	1
Cytokine release syndrome	2	2
Diabetes mellitus	2	2
Myasthenia gravis	2	1
Adrenal insufficiency	2	1
Thyrotoxicosis	2	0
Hypophysitis	1	1
Hepatitis	1	1

Multiple irAEs were observed in some patients. Adverse events were graded according to CTCAE version 5.0.

irAE, immune-related adverse event; CTCAE, Common Terminology Criteria for Adverse Events.

The baseline patient characteristics are summarized in Table 2. No significant differences were observed in age, sex, body mass index, smoking status, Eastern Cooperative Oncology Group performance status, clinical stage, histologic subtype, or programmed death-ligand 1 expression. Tumor size was larger in patients with preoperative irAEs (4.9 [4.0–5.9] cm versus 4.5 [3.1–6.2] cm), although the difference was not statistically significant (*p* = 0.216).

**Table 2:** Baseline Patient Characteristics

Variable	With irAE (n = 24)	Without irAE (n = 106)	*p* Value
Age (y)	71 [66–73]	71 [64–75]	0.524
Sex			>0.999
Male	20 (83.3)	88 (83.0)	
Female	4 (16.7)	18 (17.0)	
BMI	23.1 [20.8–25.2]	22.4 [19.9–24.5]	0.423
Smoking status			0.616
Current	7 (29.2)	26 (24.5)	
Noncurrent	17 (70.8)	80 (75.5)	
Smoking index	910 [743–1065]	800 [600–1055]	0.127
PS			>0.999
0	22 (91.7)	94 (88.7)	
≥1	2 (8.3)	12 (11.3)	
% VC	97.8 [87.5–116.2]	103.9 [94.9–113.2]	0.313
% DLCO	88.9 [85.1–90.5]	93.6 [84.2–114.2]	0.275
Tumor size (cm)	4.9 [4.0–5.9]	4.5 [3.1–6.2]	0.216
cN status			0.596
N0	7 (29.2)	24 (22.6)	
N1-2	17 (70.8)	82 (77.4)	
cStage (ninth)			0.355
II	7 (29.2)	44 (41.5)	
III	17 (70.8)	62 (58.5)	
Histology			0.417
Adenocarcinoma	10 (41.7)	45 (42.5)	
Squamous cell carcinoma	13 (54.2)	46 (43.4)	
Others	1 (4.2)	15 (14.2)	
PD-L1 expression			0.120
<1%	6 (25.0)	11 (10.4)	
1–49%	8 (33.3)	46 (43.4)	
≥50%	4 (16.7)	33 (31.1)	
Unknown	6 (25.0)	16 (15.1)	
SUV max	15.9 [9.7–18.6]	14.8 [10.4–18.1]	0.757

Values are medians [IQR] or numbers (%).

BMI, body mass index; DLCO, diffusing capacity of the lung for carbon monoxide; IQR, interquartile range; irAE, immune-related adverse event; PD-L1, programmed death-ligand 1; PS, performance status; SUV, standardized uptake value; VC, vital capacity.

Baseline laboratory data, including hematologic and tumor marker levels, are presented in Table 3. Most values, including white blood cell count, neutrophil percentage, lymphocyte percentage, monocyte percentage, C-reactive protein level, and tumor markers, were comparable between the two groups. The eosinophil fraction tended to be higher in patients with preoperative irAEs than in those without (2.3 [1.2–3.3]% versus 1.7 [0.9–2.6]%, *p* = 0.064).

**Table 3:** Laboratory Data Before Neoadjuvant Therapy

Variable	With irAE (n = 24)	Without irAE (n = 106)	*p* Value
WBC (/μL)	6820 [5825–8200]	7630 [6193–8668]	0.316
Neutrophil (%)	65.2 [58.2–70.6]	66.2 [61.9–72.6]	0.257
Lymphocyte (%)	24.5 [20.5–30.3]	22.9 [18.9–29.2]	0.479
Monocyte (%)	6.8 [5.6–8.9]	6.5 [5.2–7.6]	0.471
Eosinophil (%)	2.3 [1.2–3.3]	1.7 [0.9–2.6]	0.064
Hb (g/dL)	14.3 [12.8–14.7]	13.6 [12.3–14.7]	0.382
PLT (×10^3^/μL)	26.1 [24.4–29.3]	28.0 [23.3–32.7]	0.517
Cre (mg/dL)	0.76 [0.66–0.87]	0.79 [0.67–0.88]	0.610
ALB (g/dL)	4.0 [3.6–4.2]	3.9 [3.6–4.2]	0.410
T-Bil (mg/dL)	0.5 [0.5–0.7]	0.5 [0.4–0.7]	0.288
T-Chol (mg/dL)	181 [159–201]	186 [161–213]	0.335
CRP (mg/dL)	0.33 [0.10–1.00]	0.37 [0.14–1.91]	0.376
CEA (ng/mL)	5.9 [3.4–19.7]	4.7 [2.3–10.0]	0.420
CYFRA (ng/mL)	3.1 [1.5–5.5]	2.4 [1.6–5.5]	0.640

Values are medians [IQR].

ALB, albumin; CEA, carcinoembryonic antigen; Cre, creatinine; CRP, C-reactive protein; CYFRA, cytokeratin 19 fragment; Hb, hemoglobin; IQR, interquartile range; irAE, immune-related adverse event; PLT, platelet count; T-Bil, total bilirubin; T-Chol, total cholesterol; WBC, white blood cell count.

### Neoadjuvant Therapy and Surgical Eligibility

The details of neoadjuvant therapy are summarized in Table 4. No notable difference was observed in the selection of platinum agents between patients with and without preoperative irAEs. The distribution of the number of neoadjuvant cycles differed significantly between the groups (*p* <0.001); in patients with preoperative irAEs, 29.2% received one cycle, 12.5% received two cycles, and 58.3% completed all three cycles, whereas 92.5% of patients in those without completed all three cycles, with only 4.7% and 2.8% receiving one or two cycles, respectively. The overall completion rate of neoadjuvant therapy was significantly lower in patients with preoperative irAEs than in those without (58.3% versus 92.5%, *p* <0.001).

**Table 4:** Neoadjuvant Therapy and Surgical Eligibility

Variable	With irAE (n = 24)	Without irAE (n = 106)	*p* Value
Platinum agent			0.783
CDDP	4 (16.7)	22 (20.8)	
CBDCA	20 (83.3)	84 (79.2)	
Cycle			<0.001
1	7 (29.2)	5 (4.7)	
2	3 (12.5)	3 (2.8)	
3	14 (58.3)	98 (92.5)	
Neoadjuvant therapy completion			<0.001
Completed	14 (58.3)	98 (92.5)	
Not completed	10 (41.7)	8 (7.5)	
Surgical cancellation^a^	5 (21.7)	6 (5.8)	0.029
irAE	3	0	
Disease progression	2	3	
Patient refusal	0	3	

Values are numbers (%).

CBDCA, carboplatin; CDDP, cisplatin; IQR, interquartile range; irAE, immune-related adverse event.

a Surgical cancellation rates were calculated excluding patients who were still scheduled for surgery at the time of the data cutoff (n=1 in patients with irAEs; n=3 in those without).

Among the 24 patients who developed preoperative irAEs, 10 received corticosteroid therapy, whereas the other 14 were managed with nonsteroidal interventions, including hormonal replacement and dermatologic topical treatment. Six steroid-treated patients underwent surgery.

Surgical cancellations occurred significantly more frequently in patients with preoperative irAEs (21.7% [5/23] versus 5.8% [6/103], *p* = 0.029), excluding patients who were scheduled for surgery at the time of the data cutoff (n = 1 and n = 3, respectively). Among the five cancellations in patients with preoperative irAEs, three were because of irAEs and two were because of disease progression. Among the six cancellations in patients without preoperative irAEs, three were because of disease progression and three were because of patient refusal.

Among patients who did not complete the planned course of neoadjuvant therapy, 10 cases were observed in patients with preoperative irAEs and eight in those without. Surgery was ultimately performed in 6 and 5 patients, respectively. The median interval from the last administration of neoadjuvant therapy to surgery was 66.5 days (range, 42–140) in patients with preoperative irAEs and 55 days (range, 29–64) in those without. There was no statistically significant difference between the two groups (*p* = 0.067).

### Surgical and Pathologic Outcomes

The surgical and pathologic characteristics of the patients who underwent resection are summarized in Table 5. This included 18 patients in patients with preoperative irAEs and 97 patients without. Lobectomy was the most frequently performed procedure in both groups. The operative time was significantly longer in patients with preoperative irAEs (313 [222–386] min versus 237 [197–308] min, *p* = 0.045), and blood loss tended to be greater (172 [34–288] mL versus 65 [23–137] mL, *p* = 0.054). No significant differences were observed in the postoperative complication rates (33.3% versus 28.4%, *p* = 0.778), duration of chest drainage (3 [2–7] days versus 3 [2–5] days, p=0.628), or length of postoperative hospital stay (9 [7–11] days versus 8 [7–11] days, p=0.363). No 30-day mortality occurred in patients with preoperative irAEs, whereas one patient (1.0%) in those without died within 30 days after surgery.

**Table 5:** Surgical and Pathologic Characteristics of Patients Who Underwent Resection

Variable	With irAE (n = 18)	Without irAE (n = 97)	*p* Value
Surgical approach			0.426
Open thoracotomy	13 (72.2)	66 (68.0)	
VATS	0	10 (10.3)	
RATS	5 (27.8)	21 (21.6)	
Procedure			0.784
Exploratory thoracotomy	0	1 (1.0)	
Segmentectomy	0	2 (2.1)	
Lobectomy	16 (88.9)	82 (84.5)	
Bilobectomy	1 (5.6)	10 (10.3)	
Pneumonectomy	1 (5.6)	2 (2.1)	
Lymph nodes dissection			0.070
ND0–1	0	4 (4.1)	
ND2a–1	3 (16.7)	40 (41.2)	
ND2a–2	15 (83.3)	53 (54.6)	
Operative time (min)	313 [222–386]	237 [197–308]	0.045
Blood loss (mL)	172 [34–288]	65 [23–137]	0.054
Postoperative complications (within 30 days)	6 (33.3)	27 (28.4)	0.778
30–day mortality	0	1 (1.0)	>0.999
Chest tube duration (days)	3 [2–7]	3 [2–5]	0.628
Postoperative hospital stay (days)	9 [7–11]	8 [7–11]	0.363
Pathologic response^a^			0.157
Non–MPR	11 (61.1)	35 (36.8)	
MPR	7 (38.9)	60 (63.1)	
pCR	5 (27.8)	35 (36.8)	
Resection margin status			>0.999
R0	17 (94.4)	92 (94.8)	
R1-2	1 (5.6)	5 (5.2)	

Values are medians [IQR] or numbers (%).

IQR, interquartile range; MPR, major pathologic response; pCR, pathologic complete response; RATS, robotic-assisted thoracic surgery; VATS, video-assisted thoracic surgery.

a The number of patients varied because of missing data in patients without irAEs; analyses were performed using available data.

In patients with and without preoperative irAEs, respectively, the major pathologic response (MPR) rates were 38.9% and 63.1%, the pathologic complete response (pCR) rates were 27.8% and 36.8%, and the R0 resection rates were 94.4% and 94.8%.

### Predictive Factors for Preoperative irAEs

The analyses of potential risk factors for preoperative irAEs are summarized in Table 6. In the exploratory univariable logistic regression analysis, tumor size greater than 3.8 cm (OR, 4.42; 95% CI, 1.24–15.70; *p* = 0.022) and eosinophil fraction greater than or equal to 2.0% (OR, 2.54; 95% CI, 1.02–6.33; *p* = 0.046) were associated with the development of irAEs.

**Table 6:** Univariable Analysis of Risk Factors for Preoperative irAEs

Factor	Univariable	
	OR (95% CI)	*p* Value
Age ≥70 (vs. <70 y)	1.00 (0.40–2.44)	0.989
Male (vs. female)	1.02 (0.31–3.35)	0.970
Smoking index ≥600 (vs. <600)	3.22 (0.71–14.70)	0.131
Tumor size >3.8 cm (vs. ≤3.8)	4.42 (1.24–15.70)	0.022
cN1-2 (vs. N0)	0.71 (0.26–1.91)	0.499
cStage III (vs. II)	1.72 (0.66–4.51)	0.267
Squamous cell carcinoma (vs. adenocarcinoma)	1.82 (0.66–4.97)	0.245
PD-L1 ≥50% (vs. <50%)	0.49 (0.15–1.62)	0.244
CDDP regimen (vs. CBDCA)	0.76 (0.24–2.46)	0.652
WBC ≥7000 /μL (vs. <7000)	0.45 (0.19–1.11)	0.084
Neutrophil ≥60% (vs. <60%)	0.46 (0.18–1.19)	0.110
Lymphocyte ≥30% (vs. <30%)	2.29 (0.86–6.12)	0.099
Monocyte ≥8% (vs. <8%)	1.41 (0.52–3.79)	0.499
Eosinophil ≥2% (vs. <2%)	2.54 (1.02–6.33)	0.046
Cre ≥0.8 mg/dL (vs. <0.8)	0.65 (0.26–1.61)	0.348
ALB <4.0 g/dL (vs. ≥4.0)	0.61 (0.25–1.51)	0.287

ALB, albumin; CBDCA, carboplatin; CDDP, cisplatin; CI, confidence interval; Cre, creatinine; irAE, immune-related adverse event; WBC, white blood cell count.

## Discussion

In this multicenter retrospective study, preoperative irAEs occurred in 18.5% of the patients treated with neoadjuvant nivolumab plus chemotherapy. Patients who developed irAEs had a significantly lower rate of neoadjuvant therapy completion and a higher incidence of surgical cancellation, indicating that preoperative irAEs may substantially impact the continuity of planned perioperative treatment. Despite these challenges, curative-intent surgery was ultimately performed in 78.3% of the patients who developed preoperative irAEs, and notably, none of these operations were aborted intraoperatively. Although both MPR and pCR rates were lower than those in patients without preoperative irAEs, this outcome is likely attributable, at least in part, to the reduced number of treatment cycles in patients with preoperative irAEs rather than to irAEs themselves. Nevertheless, R0 resection was achieved in 94.4% of these patients. These findings suggest that although the development of preoperative irAEs can interfere with the intended course of neoadjuvant therapy, surgical feasibility, and perioperative safety may still be preserved in most patients with preoperative irAEs.

In the supplementary data of the CheckMate 816 trial, all-grade irAEs were reported in 19.9% of patients, which closely aligned with the frequency observed in the present study (18.5%).1 Similarly, retrospective data have documented a 15.2% incidence of grade 3 or higher irAEs.^5^ In addition, systematic reviews and meta-analyses have reported that the incidence of all-grade irAEs ranges from 16.8% to 30.4%,^6^^,^^7^ whereas that of grade 3 or higher irAEs ranges from 3.5% to 25.7%.^6^^,^^8^^,^^9^ Although variability exists across studies, these findings collectively suggest that preoperative irAEs occur in approximately 20% of patients undergoing neoadjuvant chemoimmunotherapy.

Several risk factors for the development of irAEs have been proposed in previous studies, including younger age, good performance status, high body mass index, long-term smoking history, elevated C-reactive protein levels, and high programmed death-ligand 1 expression levels.^10^^,^^11^ However, these studies have primarily focused on advanced-stage lung cancer, and evidence specific to the neoadjuvant setting for resectable NSCLC remains limited. In the present exploratory analysis, tumor size greater than 3.8 cm and eosinophil fraction greater than or equal to 2.0% at the initiation of treatment were associated with the development of preoperative irAEs in the univariable logistic regression analysis. Tumor size may reflect the tumor antigen burden and the degree of immune stimulation, which has been proposed as a mechanism contributing to excessive immune activation and the onset of irAEs in previous studies of advanced NSCLC.^12^^,^^13^ In addition to tumor size, lymph node involvement could also contribute to the overall antigen load. However, because of the limited sample size in this study, we were unable to assess the combined impact of tumor size and nodal status. Furthermore, eosinophils play a key role in Type 2 helper T-cell immune responses, facilitating autoimmune-prone inflammatory conditions through the release of cytokines, such as interleukin-5 and various chemokines. They also contribute to antigen presentation and T-cell activation, potentially amplifying the immune activation induced by immune checkpoint inhibitors.^14^, ^15^, ^16^ These findings suggest that the baseline tumor size and eosinophil fraction may serve as potential clinical indicators of the risk of irAEs in neoadjuvant settings, although further validation in larger prospective cohorts is warranted.

In the present analysis, operative time was significantly longer, and intraoperative blood loss tended to be greater in patients with preoperative irAEs. These differences, however, may not exclusively reflect the impact of irAEs themselves. Tumor-related factors, including larger tumor size or anatomically challenging locations, could also have contributed to the increased surgical complexity. Given the limited sample size, our study was not sufficiently powered to disentangle the relative effects of irAEs and tumor characteristics. Accordingly, these observations should be interpreted with caution, and future studies with larger cohorts will be required to clarify the interplay between irAEs, tumor burden, and surgical outcomes.

In the present study, 60.0% (six of 10) of patients who received systemic corticosteroids for preoperative irAEs were still able to undergo surgery, indicating that immunosuppressive management can facilitate surgical completion even after the onset of irAEs. Previous studies have reported that irAEs may be associated with favorable long-term outcomes in patients receiving immune checkpoint inhibitors.^17^, ^18^, ^19^ Accordingly, definitive resection of the primary tumor may further enhance the therapeutic efficacy. In previous retrospective studies, the R0 resection rate after neoadjuvant chemoimmunotherapy ranged from 93.5% to 98.3%,^20^^,^^21^ which is consistent with our findings. These findings support the notion that preoperative irAEs do not necessarily compromise surgical curability.

## Limitations

This study had several limitations. First, the number of patients who experienced preoperative irAEs was relatively small (18.5%, n = 24), which may have limited the statistical power to detect any subtle associations. Accordingly, the risk factor analysis was exploratory in nature and limited to univariate logistic regression, and the identified predictors should be considered hypothesis-generating. Second, as a retrospective multicenter study, heterogeneity in the definition, recognition, and grading of irAEs across institutions may have introduced variability in the classification and reporting of these events, despite efforts to align assessments with Common Terminology Criteria for Adverse Events version 5.0. This limitation may have affected the accuracy and consistency of the data. Third, although the administration of corticosteroids was recorded, detailed information regarding the dosage, duration, and timing, and the clinical response to immunosuppressive therapies was not captured in the case report forms. This limitation precluded a more nuanced assessment of how specific management strategies for irAEs may have influenced perioperative safety, surgical timing, or eligibility. In addition, data on surgical delay were not collected, and a small amount of missing data remained for the pathologic response. Future prospective studies using standardized criteria for irAE assessment and treatment are needed to clarify their clinical impact in neoadjuvant settings. Finally, long-term survival outcomes were not analyzed in this study because the follow-up period was insufficient and the data remain immature. This limitation restricts the ability to assess long-term clinical benefit. However, a follow-up program for this cohort has already been established, and future analyses are planned to clarify the impact of preoperative irAEs on long-term outcomes as the data mature.

In conclusion, in this multicenter retrospective study of patients with resectable NSCLC treated with neoadjuvant nivolumab plus chemotherapy, preoperative irAEs occurred in 18.5% of the patients and were associated with lower completion of neoadjuvant therapy and increased surgical cancellation. More importantly, surgical resection remained feasible in most patients with preoperative irAEs, and R0 resection was achieved in 94.4% of these cases. Perioperative outcomes, including complication rates and length of hospital stay, were comparable to those without irAEs. However, both the MPR and pCR rates were lower in patients with preoperative irAEs. Overall, our findings indicate that, although preoperative irAEs can interfere with the continuity of neoadjuvant chemoimmunotherapy, surgical resection remains a reasonable and achievable option with acceptable short-term outcomes.

## CRediT Authorship Contribution Statement

**Takuya Watanabe:** Conceptualization, Methodology, Validation, Formal analysis, Investigation, Resources, Data curation, Writing - original draft, Writing - review & editing, Visualization, Project administration.

**Kotaro Nomura:** Conceptualization, Investigation, Resources, Data curation, Writing - review & editing.

**Shinkichi Takamori:** Conceptualization, Investigation, Resources, Data curation, Writing - review & editing.

**Shinya Tane:** Investigation, Resources, Writing - review & editing.

**Shuta Ohara:** Investigation, Resources, Writing - review & editing.

**Hana Oiki:** Investigation, Resources, Writing - review & editing.

**Shinya Katsumata:** Investigation, Resources, Writing - review & editing. **Makoto Endo:** Investigation, Resources, Writing - review & editing.

**Satoshi Takamori:** Investigation, Resources, Writing - review & editing. **Marina Nakatsuka:** Investigation, Resources, Writing - review & editing. **Hironori Tenpaku:** Investigation, Resources, Writing - review & editing. **Ryuji Nakamura:** Investigation, Resources, Writing - review & editing.

**Hirotsugu Notsuda:** Investigation, Resources, Writing - review & editing. **Kei Namba:** Investigation, Resources, Writing - review & editing.

**Kentaro Minegishi:** Investigation, Resources, Writing - review & editing. **Masayuki Tanahashi:** Supervision, Writing - review & editing.

**Masahiro Tsuboi:** Supervision, Writing - review & editing.

**Junichi Soh:** Supervision, Writing - review & editing.

**Mototsugu Shimokawa:** Formal analysis, Validation, Writing - review & editing, Supervision.

Yasuhisa Ohde: Supervision, Writing - review & editing.

## Disclosure

Dr. Watanabe reports receiving fees for consulting, advisory, speaking, or lecture engagement with B. Braun Aesculap, Ethicon, Inc., Medtronic, PLC, MSD K.K., Chugai Pharmaceutical Co., AstraZeneca K.K., Ono Pharmaceutical Co., Konica Minolta, Inc. Dr. Nomura reports receiving fees for speaking or lecture engagement with AstraZeneca Chugai Pharmaceutical Co., Ltd., Taiho Pharmaceutical Co., Ltd., Takeda Pharmaceutical Co., Ltd., and Nipponrinshosha Co., Ltd. Dr. Ohara reports a relationship with AstraZeneca. Dr. Katsumata reports board membership with or receiving funding grants, speaking, or lecture fees from Takeda Science Foundation, Medtronic academic support, Johnson & Johnson Medical research Grant, AstraZeneca, Eli Lilly Japan K.K., COVIDIEN JAPAN Inc., and AstraZeneca. Dr. Tsuboi reports board membership with or receiving funding grants, speaking, or lecture fees from Merck & Co. Inc., MiRXES, Johnson & Johnson, Medtronic Japan, Eli Lilly Japan, Chugai Pharmaceutical, Taiho Pharma, Bristol-Myers Squibb, Ono Pharmaceutical CO., Novalis, Merck & Co., Daiichi-Sankyo, Amgen KK, Chugai Pharmaceutical CO., AstraZeneca KK. Dr. Soh reports receiving funding grants or speaking or lecture fees from Chugai Pharmaceutical, Medtronic, and CSL Behring. Dr. Ohde reports receiving speaking and lecture fees from AstraZeneca, KM biologics, and Medtronic. The remaining authors declare no conflict of interest.
